# Spontaneous rupture of a large non-parasitic liver cyst: a case report

**DOI:** 10.1186/1752-1947-4-2

**Published:** 2010-01-08

**Authors:** Lazaros Miliadis, Triantafillos Giannakopoulos, Georgios Boutsikos, Ioannis Terzis, Ioannis D Kyriazanos

**Affiliations:** 1 Department of Surgery, Naval and Veterans Hospital of Athens, Athens, Greece

## Abstract

**Introduction:**

Non-parasitic hepatic cysts are benign entities, occur rarely (5% of the population), and in the majority of cases, are asymptomatic. Cysts can cause symptoms when they become large and produce bile duct compression or portal hypertension, and also when complications such as rupture, infection or hemorrhage take place.

**Case presentation:**

We present the case of a 70-year-old Greek-Caucasian man with a large, asymptomatic and non-parasitic liver cyst that presented as an acute surgical abdominal emergency after spontaneous rupture into the peritoneal cavity.

**Conclusions:**

We present an extremely rare complication of simple liver cyst, its rupture in the free abdominal cavity, and its presentation as an acute abdomen. Large simple liver cysts should be treated with intervention at early recognition as conservative management usually results in their recurrence.

## Introduction

Recent technical advances, cost reduction and increased use of radiological imaging modalities have resulted to an increased detection of simple or non-parasitic hepatic cysts (NPHC) in approximately 1% to 5% of the general population [1].

Found more frequently in women than in men (3:1), NPHC are usually asymptomatic [1]. When they reach a substantial size, as ocurs in 5% of cases, they may become symptomatic (more commonly in women than in men at 10:1) with upper abdominal pain, bloating, nausea, vomiting and dyspnea [2]. Obstructive jaundice and portal hypertension may also occur depending on the volume and position of the cyst [3]. Complications of NPHC include hemorrhage, infection, rupture into the peritoneal cavity, the biliary tree or adjacent hollow viscus [4,5], and even acute pulmonary embolism [6].

In this report we present the case of a 70-year-old man who was admitted to our hospital with diffuse abdominal pain after the spontaneous rupture of a large non-parasitic hepatic cyst. The patient was treated through surgery wherein a wide unroofing of the cyst was performed.

## Case presentation

A 70-year-old Greek-Caucasian man was admitted to our hospital with diffuse abdominal pain of sudden onset three hours prior to his admission. The patient did not complain of nausea, vomiting or diarrhea and his temperature and arterial pressure were normal despite an elevated pulse rate (90 ppm.). His latest stool passage was blood-free and a digital rectal examination revealed nothing pathological.

During physical examination, the patient's abdomen was mildly distended with diffuse guarding and marked rebound tenderness. Abdominal sounds were diminished during auscultation.

Laboratory investigations revealed normal values for his hematocrit, hemoglobin, white blood cell count, and platelets. Renal and hepatic function tests were also normal and his blood glucose was a little elevated at 167.8 mg/dl (normal 70 to 110 mg/dl).

**Figure 1:**
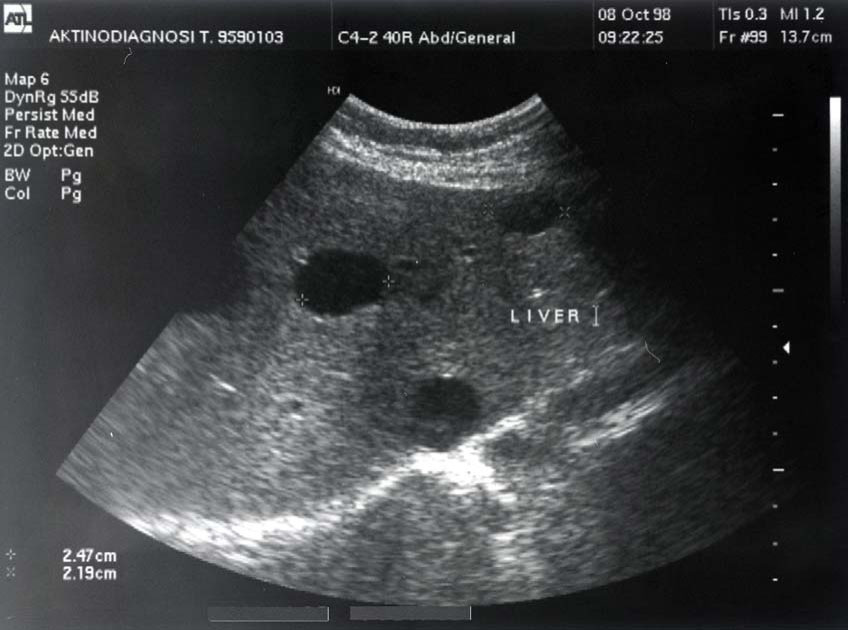
**Appearance via ultrasonography of simple liver cysts two years before the patient's latest admission**.

**Figure 2:**
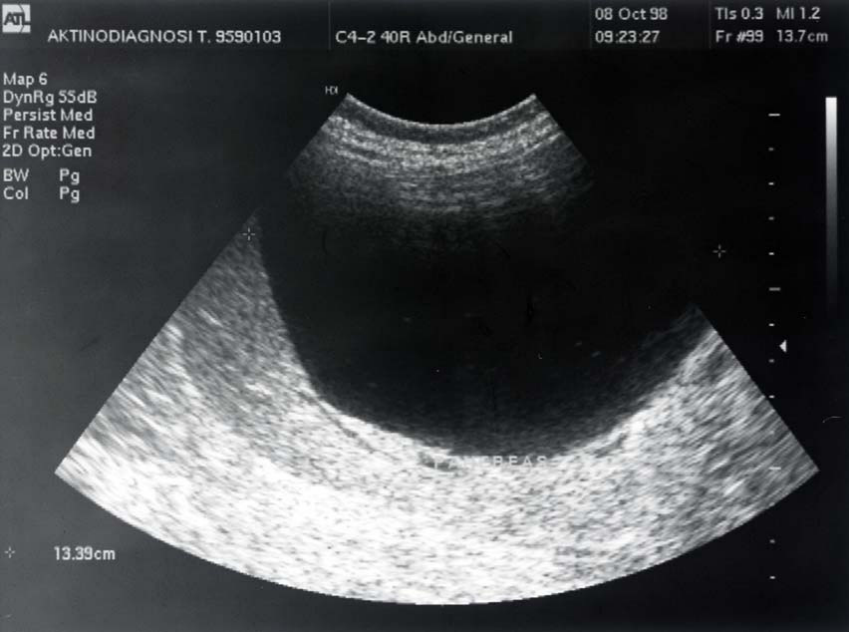
**Another view via ultrasonography of the patient's simple liver cysts two years before his latest admission**.

The patient's medical history included colonic diverticular disease, an endoscopic excision of benign rectal polyps four years prior to his presentation, and ongoing arterial hypertension and osteoporosis treatment. Ten years prior to presentation in a random ultrasound examination, the patient was found to have several simple liver cysts including two large hepatic cysts and other smaller ones. The largest cyst had a size of 13 cm. At the time, his pancreas, spleen and kidneys were normal (Figures 1 and 2). A second ultrasound examination was performed nine years after the first one (and just 13 months prior to his present admission) due to the patient being admitted after an accidental fall. A reduction in the size of the largest cyst form 13 cm to 4.6 cm and a small amount of free liquid in the patient's right abdominal fossa were identified as the only difference from the previous ultrasound report.

During the patient's present admission, there was no free air in his abdomen. An abdominal X-ray examination did not show bowel air-fluid levels. Abdominal ultrasound examination showed a significant quantity of free liquid in his abdominal cavity, around the spleen and liver, as well as in the Douglas pouch. Since the patient's general condition was deteriorating and he was already showing symptoms of paleness, sweating, increased abdominal guarding and marked rebound tenderness in the whole abdominal area, we decided to perform an exploratory laparoscopy.

The laparoscopy revealed a vast amount of opaque-yellowish peritoneal fluid occupying majority of his abdominal cavity without any obvious origin, so the operation was converted to laparotomy.

**Figure 3:**
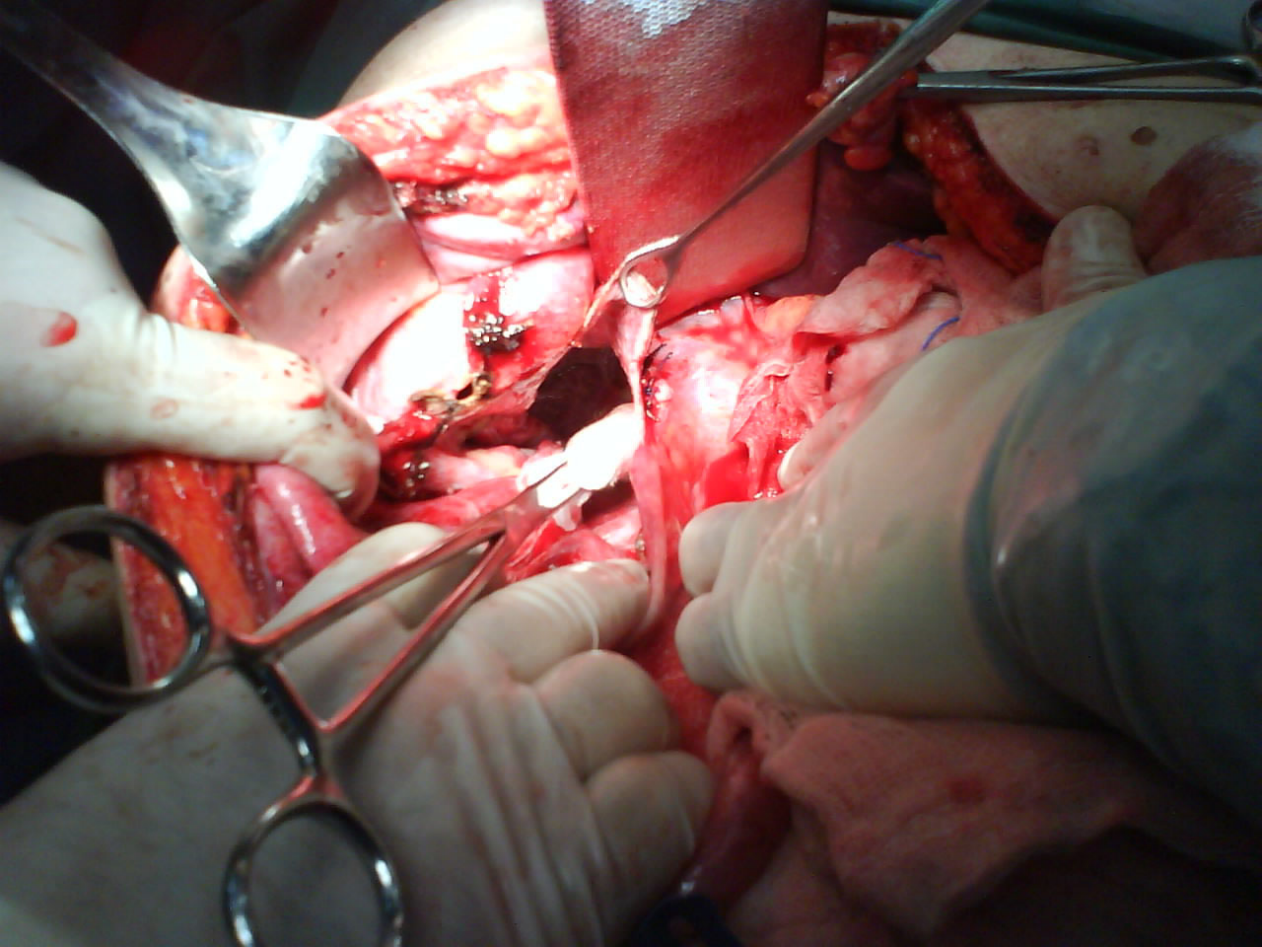
**Intraoperative view of the ruptured liver cyst located in the inferior liver surface and adjacent to the gallbladder bed**.

The exploration of the patient's abdominal cavity revealed a ruptured liver cyst that originated from the lower surface of his right liver lobe (Figure 3). Unroofing of the cyst using LigaSure to the liver parenchyma margin, plus omentoplasty and cholecystectomy, were performed as the gallbladder was part of the anterior cystic wall. Intraoperative frozen sections of multiple specimens from the cystic wall showed no evidence of malignancy, while cytology and cultures of the cystic fluid were negative. Serological tests for *Echinococcus *and tumor markers, CEA and CA 19-9, all showed negative results.

Two drains were positioned, one at the patient's cystic cavity area and the other at his Douglas pouch. The postoperative course of the patient was uneventful and three days later the drainages were removed. The patient was discharged in excellent general condition eight days after his admission.

## Discussion

Depending on the presence of an epithelial lining, liver cysts are classified as true or false. True liver cysts include congenital cysts (simple cysts and polycystic liver disease), parasitic cysts (caused by *Echinococcus *granulosis or *Echinococcus *multilocularis), neoplastic cysts (cystadenoma, cystadenocarcinoma, cystic sarcoma, squamus cell carcinoma and metastatic cancers from ovaries, colon, kidneys and pancreas) and biliary duct-related cysts (Caroli disease, bile duct duplication and peribiliary cysts). False liver cysts may be caused by spontaneous intrahepatic hemorrhage, post-traumatic hematoma, or intrahepatic biloma [7].

Differential diagnosis among several types of true liver cysts is of paramount importance because each type requires specific management. Ecchinococcal serology should be obtained in all patients with cystic liver lesions except for rare cases of cystic tumors. Ultrasonography can contribute in differential diagnosis because apart from its imaging characteristics, it can also support the performance of a cytologic diagnosis. Computed tomography (CT) with IV contrast administration is a suitable imaging modality for cyst detection, localization and sizing. It also offers significant information concerning differential diagnosis [8]. While neoplastic cysts may resemble simple cysts in CT scan, they can be usually distinguished as they often demonstrate a build-up of tissue along one wall, and/or a hypervascularity of the cyst wall [9].

On the other hand, parasitic cysts are less confusing. Compared to their non-parasitic counterparts, parasitic cysts rarely show the same type of homogenous full-of-fluid interior in CT scan, and their lumen contains daughter cysts and a considerable amount of solid debris.

Simple non-parasitic hepatic cysts are congenital and are supposedly triggered by chromosome 16 [9]. They are lined by cuboidal epithelium and arise as an aberration of bile duct development in utero. Although they are generally solitary, there may also be a simultaneous presence of more than one ("several solitary") cyst even if polycystic liver disease is absent. More recently, the presence of multiple simple liver cysts has been classified as follows: Type I, few large cysts (> 7 cm to 10 cm); Type II, multiple medium cysts (5 cm to 7 cm); and Type III, diffuse small to medium cysts (< 5 cm) [10]. Their development has a possible etiological connection to the presence of estrogens due to their increased frequency among women especially between 40 and 60 years of age [1].

The vast majority of simple hepatic cysts are asymptomatic. They can produce symptoms due to their size, anatomical localization, or when they become complicated. Most commonly, cyst enlargement can produce a sensation of foreign body, epigastric pain, nausea, vomiting and postprandial bloating [1]. Complications due to their increased size and central liver localization include obstructive jaundice, portal hypertension [3], inferior vena cava thrombosis [11], and acute pulmonary embolism [6]. Although quite rare, the related complications of these cysts can be developed due to infection, torsion [9], intracystic hemorrhage [12], or a spontaneous rupture of the cyst into the peritoneal cavity, the biliary tree or an adjacent hollow viscus such as the colon [4,5,13], which occurred in our patient.

Simple asymptomatic cysts require no treatment as they can regress spontaneously [14], especially if their diameter ranges from 2 cm to 4 cm. Larger cysts (4 cm and above) can be monitored with repeated imaging, but if the cyst remains unchanged for two years, then the monitoring may be stopped [9]. Although the majority of symptomatic and complicated cysts are not life threatening, they can significantly affect a patient's quality of life. Moreover, in cases of serious complications like infection, hemorrhage or spontaneous rupture of the cyst, special treatment must be considered. Treatment options include needle aspiration with or without injection of a sclerosing agent, internal drainage with cystojejunostomy, wide deroofing (open or laparoscopic), liver resection, and liver transplantation [14].

Percutaneous (US- or CT-guided) needle aspiration apart from therapeutic application can be used as a good therapeutic test to ascertain whether abdominal symptoms are related to the liver cyst. Although its therapeutic use is associated with high relapse rates (approximately 80% to 100%), the recurrence rate can be decreased by about 20% when percutaneous needle aspiration is combined with alcohol minocycline chloride or tetracycline chloride injection [14,15]. This method is safe and relatively noninvasive, so it can be considered as a first-line treatment for patients with high surgical risk or polycystic liver disease. However, it should be considered only after a malignant or infectious aetiology and a biliary communication have all been ruled out [15,16].

Deroofing is a definite and safe treatment for liver cysts. With few exceptions, the operation can be performed laparoscopically in 94% of reported cases [10]. Combined with argon beam coagulation and electrocoagulation for the destruction of the remaining epithelium and omental transposition flap, the laparoscopic approach resulted in 0% recurrence rate compared to 11% without omentoplasty in one study [10]. However, for hepatic cysts which are very large or in locations where laparoscopic access is not possible for the complete excision of the cyst wall (superior, posterior or deep within hepatic parenchyma), open deroofing is prudent even though the morbidity is higher. Recurrence in the case of laparoscopic deroofing ranged from 0% to 20% and morbidity rates ranged from 0% to 25% [1,14].

Roux-en-Y drainage (cystojejunostomy) has been proposed as treatment for cysts communicating with the bile duct. This operative management is yet to be proven as complications like cholangitis and sepsis that require repeated antibiotic treatment and occasional hepatic resection has been frequently reported [13].

More radical approaches like complete cyst excision and hepatectomy carry a significant morbidity (up to 50%) [2]. These approaches are poorly tolerated by elderly patients and almost unacceptable for patients presenting with benign diseases, despite the fact that the reported recurrence rates are 0% [2,16].

The spontaneous rupture of NPHC is an extremely rare complication as only 15 cases have been reported in the English literature [13]. Rupture of liver cysts may be preceded by hemorrhage which would increase the tension inside the cyst [12]. Sudden abdominal pain was the most frequent symptom and only in four cases did an acute abdomen developed [13]. Although rare (we report only the fifth known case of acute abdomen attributed to spontaneous rupture of liver cyst), this possible aetiology should be included in the differential diagnosis of an acute abdomen. Imaging modalities can lead to proper diagnosis.

This unusual complication of non-parasitic simple liver cysts may not always require surgery and may still be treated conservatively without surgical intervention when its clinical presentation is still mild [5]. This was probably the case with our patient, as the size of his preexisting huge liver cyst was found significantly reduced from 13 cm to 4.6 cm 13 months prior to his present admission. Rupture of the cyst during that time was asymptomatic. However, the fact that the patient presented with an increased cyst size during his last admission implies that conservative management of rupture liver cyst may predispose a patient to recurrence and hence the need for a surgical approach.

Because simple liver cysts normally have no communication with the biliary tree, biliary leakage should not be the case in such spontaneous ruptures. However, this does not explain the frequent development of relapsing cholangitis in patients treated with cystojejunostomy [13].

## Conclusions

Spontaneous rupture of simple liver cysts is a rare complication which can mimic acute abdomen. However, it should be included in the differential diagnosis of an acute abdomen in patients with a history of a liver cyst. Best management of ruptured NPHCs seems to be related to good exposure, wide excision of the cyst wall, and omentoplasty as conservative management can lead to reposition of the cyst wall and cyst recurrence. Laparoscopic fenestration offers the best compromise between efficacy and risk, although an open surgical approach is acceptable in difficult cases.

## Abbreviations

NPHC: non-parasitic hepatic cyst; CEA: carcinoembryonic antigen; CA: carbohydrate antigen; CT: computed tomography; US: ultrasonography.

## Consent

Written informed consent was obtained from the patient for publication of this case report and any accompanying images. A copy of the written consent is available for review by the Editor-in-Chief of this journal.

## Competing interests

The authors declare that they have no competing interests.

## Authors' contributions

LM and TG analyzed and interpreted the patient's clinical data. GB made a substantial contribution in the analysis and interpretation of the patient's history and imaging data. IT and IDK were the operating surgeons of the patient. They also made substantial contributions in the conception and design of the manuscript. LM and IDK were the major contributors in writing the manuscript. All authors read and approved the final version of the manuscript.
